# Psychosocial wellbeing of people with dementia: systematic review and construct analysis

**DOI:** 10.1017/neu.2025.10021

**Published:** 2025-06-23

**Authors:** Lena M. Hofbauer, Francisca S. Rodriguez

**Affiliations:** Research Group Psychosocial Epidemiology and Public Health, German Center for Neurodegenerative Diseases (DZNE), Greifswald, Germany

**Keywords:** Patient reported outcome measures, psychosocial functioning, psychosocial intervention, dementia, psychological well-being

## Abstract

**Objective:**

Psychosocial wellbeing is increasingly recognised as a key outcome in dementia research and care, reflecting a shift towards person-centred care and patient-reported outcome measures. However, progress is hindered by a lack of a clear and consistent definition. The present systematic review aimed to establish how previous dementia research has defined the term and how existing definitions may be unified.

**Methods:**

A systematic literature review was conducted in *PubMed*, *Embase*, and *Web of Science* using only the term ‘psychosocial’ as well as terms related to dementia in the search string. Two blinded reviewers independently conducted the abstract screening and full-text screening. Definitions used in included records were extracted and their content grouped into categories and domains. For papers presenting empirical findings, quality screening was performed using *Critical Appraisal Skills Programme* (CASP) checklists and findings were narratively summarised.

**Results:**

A total of *n* = 36 records were identified that provided a definition for psychosocial wellbeing. Conceptualizations most commonly (86 %) included emotional wellbeing, social health (64%), behavioural symptoms (44%), and subjective lived wellbeing (42%). A total of *n* = 23 records also contained empirical data, which indicated that psychosocial wellbeing may be improved by several interventions such as tailored activities and validation group therapies, among others.

**Discussion:**

The construct of ‘psychosocial wellbeing’ as currently used in dementia research predominantly incorporates emotional and subjective lived wellbeing, social health, and behavioural symptoms. This indicates an emerging consensus. To progress dementia research and care practice, it is essential that future studies use a common operationalisation.

Highlights

- The term ‘psychosocial wellbeing’ is increasingly used in dementia research and practice but definitions vary in scope.
- In this systematic review, definitions most prominently involved emotional wellbeing, social health, subjective lived wellbeing and behavioural symptoms.
- To advance person-centred dementia care and research, a clear unified definition and common measurement tools are needed.

Summations

- The review finds that researchers use a multifaceted ‘psychosocial wellbeing’ construct in dementia, which prominently encapsulates emotional health and social wellbeing.
- Researchers’ conceptualisations aligns with reports of people with dementia on their definition of wellbeing. They also reflect core outcomes to which people with dementia assign importance.
- The synthesis of included studies reporting empirical data suggests that interventions can successfully target dimensions of psychosocial wellbeing in dementia.

Considerations

- There is a paucity of clear construct definitions in the literature. In many cases indirect descriptions had to be used in the synthesis process.
- Limited empirical evidence suggests that this is a developing field of research. Accordingly, the conceptualisation identified in this review may well need to be updated in the future.

## Introduction

In the 1970s, Engel evolved the formerly biomedical model of disease to include psychological and social domains (Engel, 1977). This triggered a fundamental shift in the patient care philosophy: away from a biomedical deficit focus towards person-centred care, i.e. care that centres the needs, values, and preferences of the cared-for individual (Wade & Halligan, 2017; Tramonti *et al*., 2021). In parallel, patient-reported outcomes (PROs) and the related measurement tools (PROMs) have gained importance in research and care settings (Churruca *et al*., 2021). PROs is an umbrella term, subsuming outcomes that are based on patients’ subjective experiences rather than objective markers or clinicians’ reports. PROMs, i.e. the tools used to measure PROs, usually take the form of standardised self-report questionnaires, typically completed by the patient, sometimes by a proxy (Weldring & Smith, 2013).

The general shift towards person-centred care includes the care for people living with dementia (PwD). Interventions are now increasingly targeting PROs to ensure that person-centred dementia care truly addresses the holistic needs of those affected. A 2020 review identified 25 PROMs used in studies of PwD, seven of which were dementia-specific PROMs. The majority of these were related to either symptoms of dementia, daily functioning, or quality of life (Ayton *et al*., 2020). This echoes the values of PwD, who have identified items relating to self-managing symptoms, independency, and quality of life as core outcomes for interventions (Reilly *et al*., 2020). In other words, PwD prioritise aspects known as markers of ‘living well’ with dementia and emphasise a capabilities-focused rather than a deficit-focused approach to dementia (Moyle *et al*., 2013).

Central to this emerging view is the construct of wellbeing in dementia, which Clarke and colleagues recently described in their theoretical work. Their model, which they base on the lived experience of PwD, describes wellbeing as encompassing psychological, emotional, and social aspects. (Clarke *et al*., 2020). This echoes similar works which have de-emphasised physical aspects of wellbeing and rather focus on inner and relational aspects (e.g. Dawson *et al*., 2013; Rababa *et al*., 2023; Liu *et al*., 2023). An emphasis of psychological and social aspects of wellbeing and their interplay can be expressed using the qualified term ‘psychosocial wellbeing’ (e.g. Zulfitri *et al*., 2019).

The idea of ‘psychosocial’ wellbeing draws on theoretical traditions that integrate individual psychological factors (e.g. emotional wellbeing, self-esteem) with social factors (e.g. meaningful relationships). Kitwood’s approach to dementia care, for instance, prioritises personhood and positive social interactions (Kitwood, 1997). Complementing this, the *Adaptation-Coping Model* conceptualises psychosocial wellbeing in dementia as a dynamic process in which individuals actively engage in coping strategies to maintain a sense of competence and emotional equilibrium (Dröes, 1991; Dröes *et al*., 2017) Effective coping is supported by personal resources (e.g., resilience, self-efficacy) and external resources (e.g., social support, environmental accommodations), highlighting the importance of both psychological and social domains.

Yet, there is no clear definition of what ‘psychosocial wellbeing’ refers to and surrogate terms are also in use (e.g. ‘psychosocial health’; Singh *et al*., 2024). Establishing terminological clarity will help implement a consistent and valid operationalisation in future studies which, in turn, will advance person-centred care practice. To establish how ‘psychosocial wellbeing’ is conceptualised in dementia research, we conducted a systematic review of English scientific literature involving PwD. To get an understanding of the current state of research on psychosocial wellbeing as an outcome in dementia research, we also present the empirical evidence of the identified records. Further, comparing the research community’s understanding, as determined in review, with the perspectives of PwD synthesised in previous works (Reilly *et al*., 2020; Clarke *et al*., 2020) will reveal in how far views of researchers’ align with the lived experience of PwD.

## Methods

Methods for this systematic review follow the recommendations made in the Cochrane Handbook for Systematic Reviews of Interventions (Higgins *et al*., 2019) and in the Preferred Reporting Items for Systematic Reviews (PRISMA, Page *et al*., 2021).

### Inclusion/exclusion criteria

Criteria for inclusion/exclusion of records were determined by the Population, Intervention, Control and Outcomes (PICO) model (Miller & Forrest, 2001): (P) Population: We included studies of older adults (participants ≥60 years of age) with a diagnosis (self-reported or confirmed) of any type of dementia at any stage of severity. Studies with special groups (e.g. veterans, COVID-19, HIV patients) were excluded. (I) Interventions: We included any record of interventional and observational studies, including reviews and meta-analyses reporting on such studies. (C) Comparison: No restriction was made on whether or not a comparator was included in the record but case studies were excluded. (O) Outcomes: Any record that used the term ‘psychosocial’ and reported on an outcome described by the original author’s as relating to psychosocial wellbeing. Only English language records with abstracts were included. No restriction was made on the publication date.

### Search strategy

In order to achieve a broad inclusion of records, search terms only included the term ‘psychosocial’ and terms related to cognitive health and dementia (see Supplementary File, Table S1). No other terms were entered to avoid introducing bias into what search hits would be shown. Terms were entered in *PubMed*, *Embase*, and *Web of Science*. These specialised research search engines were chosen because they collectively ensure comprehensive coverage across biomedical (*PubMed*), clinical and pharmacological (*Embase*), and interdisciplinary (*Web of Science*) research. This maximises the likelihood of capturing both theoretical and empirical studies relevant to psychosocial wellbeing in dementia. The searches were conducted on 25^th^ of May 2023.

### Screening process

Records identified in the databases were imported into an Endnote X9 (The Endnote Team, 2013) library. Duplicates were identified via the duplicates function of the software. Subsequently, records were exported into an excel sheet for conducting first the title screening, then the abstract screening, and the full-text screening. Only records that were in alignment with the PICO criteria were included in the next screening phase. Two independent reviewers conducted abstract and full-text screenings. Disagreement between the authors’ ratings was resolved in team discussions according to the PICO criteria.

### Quality assessment

Quality assessment is used to determine how much weight can be given to specific research outputs based on their scientific rigour. Specifically, it serves the purpose of identifying sources of bias in the research methodology that may affect the reliability of findings. We did not see any ‘risk for bias’ concerning the definition of psychosocial wellbeing in the identified records. However, all studies reporting original data (i.e. excluding reviews only presenting narrative results) that were retained after full-text screening underwent quality assessment. Two raters independently completed the quality assessment using the relevant checklists (i.e. for cohort studies, RCTs, qualitative studies, or systematic reviews) of the *Critical Appraisal Skills Programme* (CASP, 2023) toolbox. Each author independently used the CASP checklists to arrive at a quality rating of high, good, or poor. Consensus was checked afterwards and any disagreements resolved in discussion. In accordance with recommendations (Higgins *et al*., 2019), records judged to be of poor quality were considered inappropriate for drawing conclusions on evidence and hence not included in the section ‘Empirical evidence in studies on psychosocial wellbeing’. The level of evidence for each included record (Levels 1–6) was determined.

### Data extraction and synthesis

Given the descriptive aim of this review, data synthesis was narrative for the definition of ‘psychosocial wellbeing’. Most records did not use the term ‘psychosocial wellbeing’ directly but instead made use of surrogate terms that included the word ‘psychosocial’ and a term that was related to wellbeing or wellbeing outcomes (see section ‘Definition of ‘psychosocial wellbeing’ in the context of dementia’), as judged by research team consensus and supported in the further analysis. From each record, one rater extracted the specific terminology used in reference to ‘psychosocial wellbeing’ as well as any explicit definitions provided by authors in a given record. This process was informed by key principles of Rodgers’ *Evolutionary Concept Analysis* (Rodgers, 1989), specifically the identification of a concept of interest, of surrogate terms, data sources, and key attributes. This method allows for pragmatic description of a construct in a given context, without becoming inflexible to developments in the future (Gunawan *et al*., 2023). The extracted information was checked by a second rater and necessary corrections made.

Subsequently, definitions provided in the included records were summarised into categories in a bottom-up process (e.g. identified mentions of ‘mood’ or ‘affect’). Thereafter, related categories were grouped into domains of psychosocial wellbeing which were labelled with descriptive umbrella terms (e.g. both ‘mood’ and ‘affect’ were subsumed under the domain of ‘emotional wellbeing’). Categories and domains were again checked by a second rater and necessary corrections made. For each of the domains, we also record the measurement tools used in the included records. Further, given the surrogate terms used in connection with ‘psychosocial wellbeing’, we were interested to see if there was systematic overlap between the domains subsumed under these terms or whether they would be mutually exclusive. Overlap would support the notion that these different terms are indeed used to describe a common underlying concept of psychosocial wellbeing. In a final step, the identified domains of psychosocial wellbeing were compared with those identified in previous works summarising PwD’s conceptualisation of wellbeing (Clarke *et al*., 2020; Reilly *et al*., 2020).

For records with empirical evidence (i.e. excluding reviews only presenting narrative results), one rater additionally extracted details on the type of publication, study design, population, dementia diagnosis (e.g. severity), intervention, outcomes, and results. Again, the extracted data was checked by a second rater and necessary corrections made. Heterogeneity between the studies was high, so that a narrative summary of empirical findings is presented. For clarity, our narrative summary groups records by level of evidence (Level 1: systematic reviews/meta-analyses of RCTs; Level 2: well-designed RCTs; Level 3: controlled trials without randomisation; Level 4: case-control or cohort studies; Level 5: systematic reviews of descriptive/qualitative studies; Level 6: single descriptive or qualitative studies; Level 7: expert opinion or narrative reviews; Melnyk & Fineout-Overholt, 2023). This ranking provides an indication of the assumed robustness of the evidence, with higher levels considered more reliable.

## Results

### Record identification

**Figure 1.**
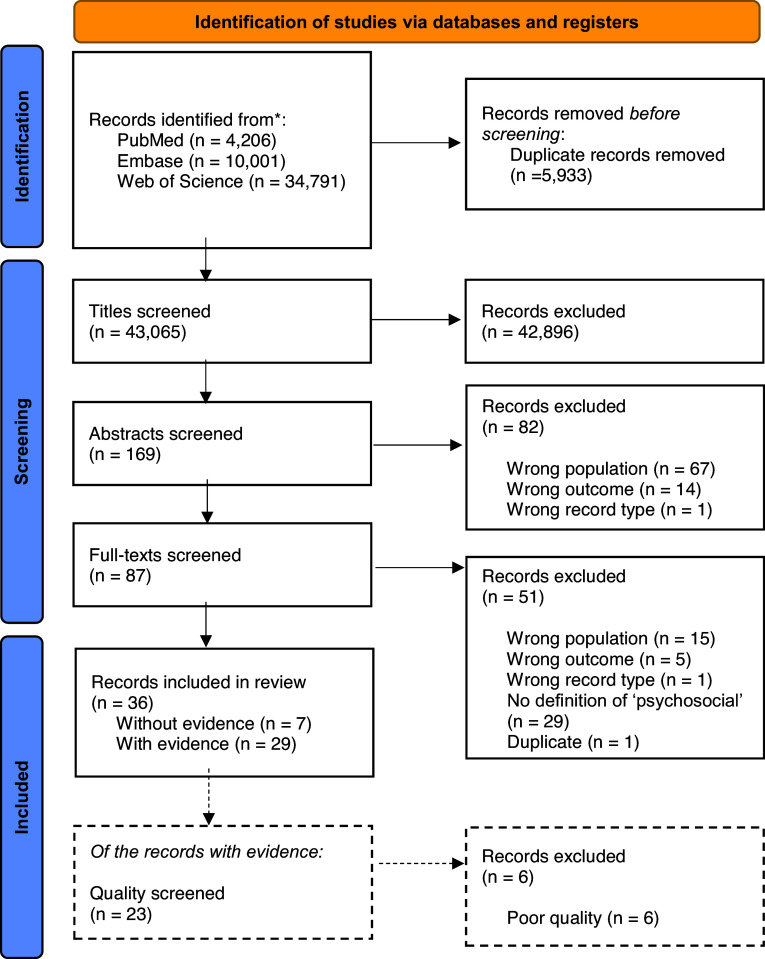
Flow chart of the screening process. Adapted from Page *et al*. (2021). *Note:* ‘with evidence’ refers to records presenting original empirical findings, ‘without evidence’ refers to records that do not present such findings.

Fig. 1 provides details on the selection process. The search in the databases identified a total of *n* = 43,065 records (without duplicates). After removing records with titles that did not match the PICO criteria (e.g., on children, schizophrenia, HIV, or cancer patients), a total of *n* = 169 abstracts were screened. Of the abstracts, only *n* = 87 matched the PICO criteria. These records underwent full-text screening. Excluding records with the wrong population, outcome, or no definition of ‘psychosocial wellbeing’, left a total of *n* = 36 records. Four of these records were narrative reviews, *n* = 15 systematic reviews, and *n* = 17 empirical studies. Publication years ranged between 1997 and 2023. These records were used to derive the definition of ‘psychosocial wellbeing’ in the context of dementia (section ‘Definition of ‘psychosocial wellbeing’ in the context of dementia’). Screening the reference list of the included studies, we were not able to identify any additional relevant records.

Among the *n* = 36 records, *n* = 32 contained empirical data. All records were used to identify definitions (section ‘Definition of ‘psychosocial wellbeing’ in the context of dementia’). Before summarising available evidence (section ‘Empirical evidence in studies on psychosocial wellbeing’), some exclusions were made. We could not use *n* = 3 records as they did not investigate a psychosocial outcome (Cieza *et al*., 2015b; Van der Steen *et al*., 2017) or did not provide findings specifically for dementia (Sabariego *et al*., 2015). We also did not use another *n* = 6 records as they received a poor quality rating in the quality assessment (Vespa *et al*., 2002; Sidani *et al*., 2012; Kok *et al*., 2013; Ausserhofer *et al*., 2016; Yen & Lin, 2018; Rababa *et al*., 2023). Central reasons for poor quality ratings of reviews were unclear inclusion criteria and shortcomings in their quality assessment processes. For empirical studies the main issues were poor reporting on study design and statistical results. A total of *n* = 23 records with good or high quality were retained for the narrative summary of empirical findings.

### Definition of ‘psychosocial wellbeing’ in the context of dementia

**Figure 2.**
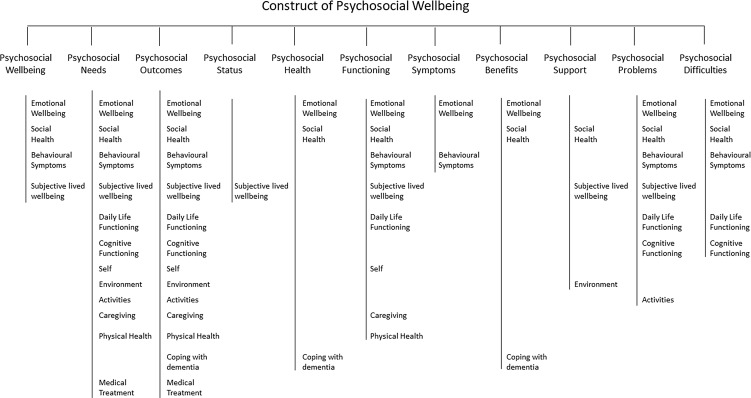
Hierarchical chart representing the terminology used related to psychosocial wellbeing and the domains subsumed under each term.

Of the *n* = 36 records used to derive the definition of ‘psychosocial wellbeing’ *n* = 4 publications used the term ‘psychosocial wellbeing’. Other records used alternative terminology, *n* = 13 ‘psychosocial outcomes’, *n* = 5 ‘psychosocial needs’, *n* = 4 ‘psychosocial functioning’, *n* = 3 ‘psychosocial difficulties’, and *n* = 2 ‘psychosocial problems’. The terms ‘psychosocial status’, ‘psychosocial health’, ‘psychosocial benefits’, ‘psychosocial support’, and ‘psychosocial symptoms’ were each used by *n* = 1. Terms used are shown in Fig. 2. Only one record provided a specific definition, ‘psychosocial health (…) consist of psychological health (…), emotional health (…) and social health (…), social and emotional wellbeing, social reintegration and psychological adjustment’ (p.188, Lakhani *et al*., 2019). Three records defined psychosocial wellbeing through the measurement that was used: *Multidimensional Observation Scale for Elderly Subject*s (MOSES) (Kok *et al*., 2013), *Modified Interaction Behavior Measure* (MIBM) and the *London Psychogeriatric Rating Scale* (Sidani *et al*., 2012), and the *Cohen-Mansfield Agitation Inventory* (CMAI) (Vespa *et al*., 2002). All other records gave indirect definitions by listing example for what was considered to be relevant to ‘psychosocial wellbeing’, e.g. ‘depression/anxiety, irritability, withdrawal, disorientation’ (p.300, Watson *et al*., 1998). Definitions can be found in the Supplementary File, Table S2.

From the direct and indirect definitions provided in the papers, we identified a total of 13 domains of psychosocial wellbeing. As Fig. 2 shows, there is substantial overlap in the domains subsumed under these different surrogate terms, without any evident systematic differences that would allow for differentiation. Thus, as determined in consensus meetings, we assume that surrogate terms such as ‘psychosocial needs’, ‘psychosocial functioning’, ‘psychosocial difficulties’, ‘psychosocial problems’ indeed all refer to concepts on the spectrum of psychosocial wellbeing, with ‘problems’ and ‘difficulties’ reflecting the absence of well-being.

Domains most commonly referred to were emotional wellbeing (86%) and social health (64%), behavioural symptoms (44%), and subjective lived wellbeing (42%). Most domains comprised several categories. For instance, papers making reference to emotional wellbeing considered 13 categories, of which the most commonly named were depression (*n* = 12), mood (*n* = 9), anxiety (*n* = 9), affect (*n* = 8), and emotional health (*n* = 6). Papers dealing with social health comprised four categories, namely social interaction (*n* = 14), social integration (*n* = 8), relationship quality (*n* = 5), and personal attention (*n* = 1). For a detailed breakdown of all domains and categories see Table 1.

**Table 1.** Psychosocial wellbeing domains and sub-categories identified in the present review compared with domains identified in previous syntheses

Domains of Psychosocial Wellbeing Construct with Sub-Categories (n of studies, % of)	Matching Core Outcome Item(s) (with Domain(s), Reilly *et al*., 2020)	Wellbeing in Dementia Domain(s) (with Theme(s), Clarke *et al*., 2020)
Emotional wellbeing (*n* = 31, 86%) (Toseland *et al*., 1997; Watson *et al*., 1998; Clark *et al*., 2004; Brandt *et al*., 2005; Damianakis *et al*., 2010; Zimmerman *et al*., 2013; Kok *et al*., 2013; Dawson *et al*., 2013; Bowen *et al*., 2014; Sabariego *et al*., 2015; Cieza *et al*., 2015a; Resnick and Galik, 2015b; Ausserhofer *et al*., 2016; Hermans *et al*., 2017; Schmidt *et al*., 2018; Yen & Lin, 2018; Lee *et al*., 2019; Lakhani *et al*., 2019; Feast *et al*., 2020; Fauth *et al*., 2020; Brancatisano *et al*., 2020; Bourne *et al*., 2021; Wang *et al*., 2021; Ha *et al*., 2021; Kumar & Salinas, 2021; Shim *et al*., 2021; Lassell *et al*., 2022; Timmons and Fox, 2023; Shoesmith *et al*., 2023; Möhler *et al*., 2023; Rababa *et al*., 2023) Depression (*n* = 12) Mood (*n* = 9) Affect (*n* = 8) Anxiety (*n* = 9) Emotional health (*n* = 6) Stress (*n* = 4) Safety (*n* = 3) Emotional bonding (*n* = 1) Comfort (*n* = 1) Embarrassment (*n* = 1) Hopelessness (*n* = 1) Psychological health (*n* = 4) Worry (*n* = 1)	Having a laugh (Quality of life)	Positive States (Emotional Well-Being)
Social health (*n* = 23, 64%) (Vespa *et al*., 2002; Clark *et al*., 2004; Damianakis *et al*., 2010; Sidani *et al*., 2012; Kok *et al*., 2013; Sabariego *et al*., 2015; Cieza *et al*., 2015a; Cieza *et al*., 2015b; Ausserhofer *et al*., 2016; Hermans *et al*., 2017; Van der Steen *et al*., 2017; Schmidt *et al*., 2018; Lee *et al*., 2019; Lakhani *et al*., 2019; Feast *et al*., 2020; Brancatisano *et al*., 2020; Bourne *et al*., 2021; Wang *et al*., 2021; Ha *et al*., 2021; Shim *et al*., 2021; Timmons and Fox, 2023; Möhler *et al*., 2023; Rababa *et al*., 2023) Social interaction (*n* = 15) Social integration (*n* = 8) Relationship quality (*n* = 5) Social health (*n* = 2) Personal attentions (*n* = 1)	Importance of relationships (Friendly neighbourhood and home),Communication (Friendly neighbourhood and home), Feeling valued and respected by others (Friendly neighbourhood and home)	Connection and Belonging (Social Well-Being)
Behavioural symptoms (*n* = 16, 44%) (Toseland *et al*., 1997; Watson *et al*., 1998; Zimmerman *et al*., 2013; Kok *et al*., 2013; Cieza *et al*., 2015a; Resnick and Galik, 2015b; Cieza *et al*., 2015b; Ausserhofer *et al*., 2016; Feast *et al*., 2020; Bourne *et al*., 2021; Shim *et al*., 2021; Lassell *et al*., 2022; Timmons and Fox, 2023; Shoesmith *et al*., 2023; Möhler *et al*., 2023; Rababa *et al*., 2023) Agitation (*n* = 9) Behavioural problems (*n* = 7) Sleeping problems (*n* = 4) Aggression (*n* = 4) Withdrawal (*n* = 3) Disorientation (*n* = 3) Apathy (*n* = 3) Hallucinations (*n* = 2) Wandering (*n* = 1) Vitality (*n* = 1) Fatigue (*n* = 2)	Apathy/indifference (Self-managing dementia symptoms)	
Subjective lived wellbeing (*n* = 15, 42%) (Clark *et al*., 2004; Zimmerman *et al*., 2013; Dawson *et al*., 2013; Ausserhofer *et al*., 2016; Hermans *et al*., 2017; Van der Steen *et al*., 2017; Yen & Lin, 2018; Feast *et al*., 2020; Bourne *et al*., 2021; Wang *et al*., 2021; Shim *et al*., 2021; Wang *et al*., 2022; Shoesmith *et al*., 2023; Möhler *et al*., 2023; Rababa *et al*., 2023) Quality of life (*n* = 11) Wellbeing (*n* = 5) Meaningful living (*n* = 5) Life satisfaction (*n* = 3) Quality of dying (*n* = 1)	Feeling safe and secure (Friendly neighbourhood and home)	Valuing Life (Life Satisfaction)
Daily life functioning (*n* = 10, 28%) (Toseland *et al*., 1997; Zimmerman *et al*., 2013; Bowen *et al*., 2014; Sabariego *et al*., 2015; Ausserhofer *et al*., 2016; Yen & Lin, 2018; Lee *et al*., 2019; Wang *et al*., 2021; Timmons & Fox, 2023; Möhler *et al*., 2023) Autonomy (*n* = 8) Capabilities (*n* = 3)		
Cognitive functioning (*n* = 9, 25%) (Clark *et al*., 2004; Damianakis *et al*., 2010; Sabariego *et al*., 2015; Cieza *et al*., 2015b; Ausserhofer *et al*., 2016; Yen and Lin, 2018; Lee *et al*., 2019; Feast *et al*., 2020; Timmons and Fox, 2023) Memory function (*n* = 6) Alertness (*n* = 3) Cognitive capacity (*n* = 2)	Alertness (Self-managing dementia symptoms), Understanding time and place (Self-managing dementia symptoms)	
Self (*n* = 8, 22%) (Damianakis *et al*., 2010; Hermans *et al*., 2017; Schmidt *et al*., 2018; Yen & Lin, 2018; Lee *et al*., 2019; Wang *et al*., 2021; Shim *et al*., 2021; Timmons & Fox, 2023) Self-esteem (*n* = 4) Sense of self (*n* = 3) Self-efficacy (*n* = 2) Self-determination (*n* = 1)	A sense of who you are (Quality of life)	Positive Sense of Self (Psychological Wellbeing), Agency and Purpose (Psychological Wellbeing)
Environment (*n* = 6, 17%) (Damianakis *et al*., 2010; Bowen *et al*., 2014; Van der Steen *et al*., 2017; Schmidt *et al*., 2018; Lee *et al*., 2019; Timmons and Fox, 2023) Living environment (*n* = 3) Territoriality (*n* = 2) Adapting to stimuli (*n* = 1)		
Physical health (*n* = 5, 14%) (Kok *et al*., 2013; Bowen *et al*., 2014; Yen & Lin, 2018; Shim *et al*., 2021; Möhler *et al*., 2023) Physical health (*n* = 5)	Vision and hearing (Quality of life), Hygiene and comfort (Quality of life), Stability (Quality of life)	
Caregiving (*n* = 5, 14%) (Bowen *et al*., 2014; Wang *et al*., 2021; Ha *et al*., 2021; Shim *et al*., 2021; Möhler *et al*., 2023) Caregiver burden (*n* = 4) Care network (*n* = 1) Caregiver education (*n* = 1)		
Activities (*n* = 5, 14%) (Zimmerman *et al*., 2013; Ausserhofer *et al*., 2016; Feast *et al*., 2020; Timmons & Fox, 2023; Möhler *et al*., 2023) Engagement in activities (*n* = 6)	Meaningful activities (Independence)	
Coping with dementia (*n* = 4, 11%) (Clark *et al*., 2004; Lakhani *et al*., 2019; Brancatisano *et al*., 2020; Wang *et al*., 2021) Adaptation (*n* = 3) Acceptance (*n* = 2)		Agency and Purpose (Psychological Wellbeing), Going Beyond (Psychological Wellbeing)
Medical treatment (*n* = 4, 11%) (Clark *et al*., 2004; Zimmerman *et al*., 2013; Bowen *et al*., 2014; Möhler *et al*., 2023) Medication use *(n* = 2) Use of restraints (*n* = 1) Service satisfaction (*n* = 1) Service availability (*n* = 1)		

A range of measurement instruments were used in the context of each psychosocial wellbeing domain. For instance, emotional wellbeing was commonly assessed using the *Center for Epidemiological Studies Depression scale* (CES-D) (Clark *et al*., 2004; Dawson *et al*., 2013; Shim *et al*., 2021), the MOSES (Toseland *et al*., 1997; Watson *et al*., 1998; Kok *et al*., 2013), *Geriatric Depression Scale* (GDS) (Ha *et al*., 2021; Shim *et al*., 2021; Shoesmith *et al*., 2023), or the *Palliative Care Outcome Scale* (POS) (Brandt *et al*., 2005; Hermans *et al*., 2017). Around half of the instruments used in the emotional wellbeing domain were self-report instruments, the other half used proxy-report. Social health was assessed either as part of a comprehensive assessment such as the POS (Hermans *et al*., 2017; Timmons & Fox, 2023) and *The Nurses’ Observation Scale for Geriatric Patients* (NOSGER) (Wang *et al*., 2021) or via specific measures of social interaction/ integration like the *Index of Social Engagement* (Möhler *et al*., 2023) and the *Mutuality scale* (Ha *et al*., 2021). The majority of instruments in this domain were proxy-report instruments. The most commonly used instrument in the context of behavioural symptoms was the *Cohen-Mansfield Agitation Inventory* (CMAI) (Toseland *et al*., 1997; Watson *et al*., 1998; Kok *et al*., 2013; Resnick & Galik, 2015a; Wang *et al*., 2022; Möhler *et al*., 2023; Shoesmith *et al*., 2023). The majority of instruments on behavioural symptoms were proxy-report instruments. For subjective lived wellbeing the most popular instrument was the *Quality of Life - Alzheimer’s Disease Scale* (QoL-AD) (Dawson *et al*., 2013; Bourne *et al*., 2021; Shim *et al*., 2021; Wang *et al*., 2021; Wang *et al*., 2022; Möhler *et al*., 2023; Shoesmith *et al*., 2023). The majority of instruments in the subjective lived wellbeing domain used self-report. An overview of measurement instruments used and in the context of each domain can be found in the Supplementary File, Table S3.

### Comparing the researchers’ definitions with syntheses of PwD reports

The domains of ‘psychosocial wellbeing’ identified under ‘Definition of ‘psychosocial wellbeing’ in the context of dementia’ partially overlap with (1) the previously published domains in the core outcome set as intervention outcomes relevant to PwD (Reilly *et al*., 2020), and (2) the previously published wellbeing domains in the previous review of PwD reports on wellbeing (Clarke *et al*., 2020), see Table 1 for the comparison. Notably, the present review also identified domains that neither of the comparison syntheses listed (i.e., Daily life functioning, environment, caregiving, medical treatment), however, as only very few studies included them, we would consider them less relevant.

### Empirical evidence in studies on psychosocial wellbeing

A total of *n* = 23 records had empirical data of good and high quality (see Supplementary Table S4). Evidence from meta-analyses (Level 1 evidence) indicates that resilience interventions could improve quality of life (Wang *et al*., 2021), tailored activities could improve agitation (Möhler *et al*., 2018), and mind-body-therapies/mindfulness could improve cognition and possibly also depression and quality of life (Shim *et al*., 2021; Wang *et al*., 2022). Level 2 and 3 evidence indicates that telephone care consultation may improve depression, relationship strain, feelings of embarrassment, and coping difficulties (Clark *et al*., 2004), daily rocking chair therapy may reduce anxiety/depression (Watson *et al*., 1998), life review programmes may increase social interactions, specifically talkativeness (Ha *et al*., 2021), and validation group therapy may reduce aggressive behaviour and depression (Toseland *et al*., 1997). Level 4 and 5 evidence suggests that dyadic art interventions (Bourne *et al*., 2021), animal-assisted and robotic animal-assisted interventions (Shoesmith *et al*., 2023), engaging with the natural environment (Lakhani *et al*., 2019), pleasant sensory stimulation (Zimmerman *et al*., 2013), individualised care (Zimmerman *et al*., 2013), function-focused care (Lee *et al*., 2019) and any hospice interventions (Lassell *et al*., 2022), may be associated with benefits for psychosocial wellbeing.

Level 6 studies show associations of positive staff interactions (Fauth *et al*., 2020), multimedia biography screenings (Damianakis *et al*., 2010), as well as role captivity, physical health strain, and difficulties in daily life (Dawson *et al*., 2013) with emotional wellbeing. Moreover, they identified needs (e.g. assistance with driving, administering medication, managing finances, shopping, Bowen *et al*., 2014; food intake, physical activity, personal attention, self-determination, Schmidt *et al*., 2018) of PwD. Compared to other palliative patients, PwD seem to have higher needs relating to support (Brandt *et al*., 2005) as well as life worthwhile and self-worth (Hermans *et al*., 2017).

## Discussion

This review aimed to identify studies relating to ‘psychosocial wellbeing’ in dementia and synthesise what the general understanding of this construct is in dementia research. We found that psychosocial wellbeing has so far rarely been explicitly defined by researchers who make reference to it in their work. Researchers most commonly used examples to describe it. For the majority of authors, psychosocial wellbeing encompasses emotional wellbeing (86%) and social health (64%), and, for more than one third of the authors, also behavioural symptoms (44%) and subjective lived wellbeing (42%). Accordingly, we come to the understanding that, in the view of the research community, psychosocial wellbeing describes the subjective lived and emotional wellbeing of a person in dementia research, including no or low levels of behavioural symptoms, together with good social interactions. In the records reviewed, researchers subsume subjective lived wellbeing, including quality of life, as aspects of ‘psychosocial wellbeing’. Our findings therefore echo previous observations of wellbeing being understood as a more comprehensive construct than quality of life (Clarke *et al*., 2020).

Comparing the domains identified in this review with those identified in a previous wellbeing in dementia review by Clarke *et al*. (2020), we observed substantial overlap. That is, the perspective of PwD on wellbeing is echoed in the understanding of the research community. Importantly, both reflect the core outcomes identified by PwD as preferred intervention endpoints. This indicates that the construct of psychosocial wellbeing is aligned with the values of those directly affected by dementia, in accordance with the priorities of person-centred care. Thus, the construct is worth pursing further. However, as ‘psychosocial wellbeing’ as understood by the research community appears to have a particularly broad scope, researchers will be required to bear in mind its high dimensionality. While future research may be able to refine the construct and narrow down important domains, presently, it is indicated to refrain from using one or two domain scores as supposedly indicative of psychosocial wellbeing (Ruggeri *et al*., 2020).

The four major aspects of psychosocial wellbeing, as identified in this review (emotional wellbeing, social health, behavioural symptoms, and subjective lived wellbeing), were assessed mostly via the CMAI, QOL-AD, depression scales (such as the CES-D or GDS), and some form of indicator of social participation or relationships. Using a combination of those could reflect the broad spectrum of psychosocial wellbeing. Adding scales that encompass further symptoms such as the MOSES (e.g., self-care, disorientation, irritability, withdrawal), the MIBM (e.g., personal attending, relaxation, calmness), the POS (e.g., pain, sharing, self-worth), or the PARADISE data collection protocol (e.g., mental functions, difficulties in activities/ participation) could provide further information on the psychosocial wellbeing of patients. Yet, it is not yet certain what instrument choices reflect PwD’s psychosocial wellbeing best. On one hand, evaluating the psychometric qualities of these instruments was beyond the scope of this research. Existing reviews report diverging quality (see e.g. Algar *et al*., 2016; Ellis-Smith *et al*., 2016; Clarke *et al*., 2020), which can be used to inform instrument selection. On the other hand, standardised measures may not always be able to capture complex multidimensional constructs fully and fall short in capturing, for example, embodied-embedded, reflective, and socially-embedded self-aspects. This is particularly true for PwD who, due to the progression of dementia, may no longer be capable of comprehensive self-report. Observational measures may be an important supplement (Algar *et al*., 2016).

While the choice of the instrument should be with the researcher, especially given constraints concerning language and licences, it may be useful to have a tool that assesses all dimensions of psychosocial wellbeing as currently no such comprehensive assessment exists. Developing such a tool could involve a multi-stage process in which experts evaluate and refine the tool, e.g. in a Delphi consensus process (e.g. Nejati *et al*., 2025). This consensus process should preferably be capabilities-focused instead of deficit-focused. As it stands, many of the tools used in the reviewed psychosocial wellbeing records are deficit-focused. For instance, in the emotional wellbeing domain, we observed a clear focus on pathology such as depression and anxiety. Going forward, to uphold the goals of person-centred care and reflect the wishes of PwD, it would be important to reframe domains in a capabilities-focused angle (Moyle *et al*., 2013; Reilly *et al*., 2020).

If a common tool was used, studies would be comparable and it would be possible to conduct meta-analyses. Presently, this was not possible given a high heterogeneity of studies and only a small body of adequate empirical research (*n* = 23). In our narrative review, findings suggested that resilience interventions, tailored activities, and mind-body therapies may improve some aspects of psychosocial wellbeing, warranting further investigation. Initial positive indication was also seen for other approaches (e.g. telephone care consultation, validation group therapy). To get a more thorough state of the art and identify feasible avenues for further intervention research, a subsequent review for each aspect of psychosocial wellbeing (e.g. emotional wellbeing, social health) may be conducted.

A strength of this review is its broad inclusion strategy, capturing diverse perspectives on psychosocial wellbeing. It represents the first attempt to conceptualise the construct of psychosocial wellbeing in the dementia context. Findings have practical relevance for future research and clinical practice. However, our results only apply to English-language records. This was a necessary restriction to make, given that our intent was to analyse the concept behind the specific term of ‘psychosocial wellbeing. Overall, it is important to note that the current results cannot be generalised beyond the existing data analysed and will have to be updated with time.

## Conclusion

The construct of ‘psychosocial wellbeing’ as currently used in research predominantly incorporates emotional and subjective lived wellbeing, including behavioural symptoms, and social health. This review thus was able to identify an emerging consensus in the research community. Moreover, the current understanding of the construct aligns well with PwD’s view on wellbeing and with their preferences for core outcomes in intervention trials. This underlines that pursuing further research in this area will help improve person-centred care. To gain a more developed notion of the psychosocial wellbeing construct, it will be necessary for future research to carefully consider how it should be operationalised. To do its multi-faceted nature justice, future studies would be well-advised to consider a broad spectrum of outcome measures, prioritising those that represent the key domains.

## Supporting information

Hofbauer and Rodriguez supplementary material Hofbauer and Rodriguez supplementary material

## Supplementary material

The supplementary material for this article can be found at https://doi.org/10.1017/neu.2025.10021.
