# Supplementary material for: Psychosocial wellbeing of people with dementia: systematic review and construct analysis
Source: Acta Neuropsychiatr. 2025 Jun 23;37:e71. doi: 10.1017/neu.2025.10021 (PMC13130359; doi:10.1017/neu.2025.10021)
Supplement: Hofbauer and Rodriguez supplementary material [file S0924270825100215sup001.docx]

Supplementary File to the Article:

**“Psychosocial wellbeing of people with dementia: Systematic review and construct analysis”**

Lena M. Hofbauer, M.Sc. (ORCID ID: 0000-0003-1789-711X)^a^, PD Francisca S. Rodriguez, PhD (ORCID ID: 0000-0003-2919-5510)^a†^

^a^ Research Group Psychosocial Epidemiology and Public Health, German Center for Neurodegenerative Diseases (DZNE), Site Rostock/Greifswald, Ellernholzstr 1-2, 17489 Greifswald, Germany

**Table S1.** Search terms and filters used in the literature databases.

| Database | Search string | Filters | Results |
| --- | --- | --- | --- |
| Pubmed | (psychosocial[Title/Abstract]) AND (((((((((((((((((((((("cognitive impairment"[Title/Abstract]) OR ("cognitive functioning"[Title/Abstract])) OR (dementia[Title/Abstract])) OR (Alzheime*[Title/Abstract])) OR (neurocognitive[Title/Abstract])) OR (neuroepidemi*[Title/Abstract])) OR ("old age"[Title/Abstract])) OR ("older adults"[Title/Abstract])) OR (elderly[Title/Abstract])) OR (demented[Title/Abstract])) OR (aging[Title/Abstract])) OR (ageing[Title/Abstract])) OR ("cognitive problems"[Title/Abstract])) OR ("cognitive decline"[Title/Abstract])) OR ("cognitive status"[Title/Abstract])) OR (neurologi*[Title/Abstract])) OR (neuropsycholog*[Title/Abstract])) OR ("memory problems"[Title/Abstract])) OR ("impaired memory"[Title/Abstract])) OR ("memory impairments"[Title/Abstract])) OR ("memory impairment"[Title/Abstract])) OR ("memory loss"[Title/Abstract])) | Abstract, Classical Article, Clinical Study, Clinical Trial, Comparative Study, Consensus Development Conference, Controlled Clinical Trial, Evaluation Study, Meta-Analysis, Observational Study, Practice Guideline, Pragmatic Clinical Trial, Randomized Controlled Trial, Review, Systematic Review, Twin Study, English | 4,206 |
| Embase | (psychosocial) AND ('cognitive impairment' OR 'cognitive functioning’ OR 'dementia' OR alzheime* OR neurocognitive OR neuroepidemi* OR 'old age' OR 'older adults' OR elderly OR demented OR aging OR ageing OR 'cognitive problems' OR 'cognitive decline' OR 'cognitive status' OR neurologi* OR neuropsycholog* OR 'memory problems' OR 'impaired memory' OR 'memory impairments' OR 'memory impairment' OR 'memory loss') | 'human'/de OR 'meta analysis'/de OR 'methodology'/de OR 'model'/de OR 'practice guideline'/de OR 'systematic review'/de) AND ([aged]/lim OR [middle aged]/lim OR [very elderly]/lim) AND ('article'/it OR 'review'/it | 10,001 |
| Web of Science | (psychosocial) AND (((((((((((((((((((((("cognitive impairment") OR ("cognitive functioning")) OR (dementia)) OR (Alzheime*)) OR (neurocognitive)) OR (neuroepidemi*)) OR ("old age")) OR ("older adults")) OR (elderly)) OR (demented)) OR (aging)) OR (ageing)) OR ("cognitive problems")) OR ("cognitive decline")) OR ("cognitive status")) OR (neurologi*)) OR (neuropsycholog*)) OR ("memory problems")) OR ("impaired memory")) OR ("memory impairments")) OR ("memory impairment")) OR ("memory loss")) (Abstract) and Article or Review Article (Document Types) and Article or Review Article (Document Types) and English (Languages) | Document Types: Article or Review Article. Document Types: Article or Review Article. Languages: English. | 34,791 |

**Table S2.** Definitions of ‘psychosocial’ wellbeing provided by authors in the included records.

| Term used | Publication | Definition given (page) |
| --- | --- | --- |
| Psychosocial needs | Bowen et al., 2014 | “care network, living situation, safety, physical state, level of independence, activities of daily living (ADLs) and instrumental activities of daily living (IADLS), caregiver education, and available resources and services” (p.668) |
|  | Lee et al., 2019 | “basic psychosocial needs of residents include territoriality, autonomy, communication, personal identity, self-esteem, cognitive understanding, safety, and security” (p.9) |
|  | Schmidt et al., 2018 | “psychosocial needs were classified as follows: adaptation of stimuli, communication, personal attention, participation, familiarity and safety, self-determination” (p.257) |
|  | Timmons and Fox, 2023 | “Psychosocial needs: Neuropsychiatric symptoms (…) are sometimes referred to as Behavioral and Psychologic Symptoms of Dementia (or BPSD) (…), behavior is understood to manifest unmet needs, or a response to a perceived threat“, “such as the assessed PIECES framework (Physical cause, Intellectual capacity, Emotional health, Capabilities, Environment, Social self) and the ABC behavioral assessment tool (Antecedent, Behavior Consequence) ” (p. 84), “Anxiety, Appearing depressed, Hallucinations, Agitation, Apathy/loss of interest, Walking about (wandering), Sleeping problems, Ability to interact with others, Ability to enjoy activities” (Table 6.1, p.84) |
|  | Hermans et al., 2017 | “needs in psychosocial and spiritual domains, such as patient anxiety, support, finding life worth living and self-worth” (p.1501) |
| Psychosocial outcomes | Resnick and Galik, 2015a | “psychosocial outcomes (mood, agitation, apathy)” (p.274) |
|  | Bourne et al., 2021 | “psychosocial outcomes refers to any reported or observed effects on emotions, well-being, QOL [quality of life], behaviour or forming and maintaining connections with others” (p.1634) |
|  | Damianakis et al., 2010 | “been amazing (…) love watching (…) enjoy” (p.29), “impact on reminiscing and (…) emotions” (p.29), “impact on patterns on communication and social interactions” (p.29), “effects on (…) memory and mood” (p.29), “ effects on (…) memory and emotion” (p.30), “impact on (…) sense of self” (p.30) |
|  | Yen and Lin, 2018 | “subjective psychosocial measurements, including the Geriatric Depression Scale-Short Form and Self-esteem, Life Satisfaction, and Health Perception Scales, or objective performance assessments (e.g., MMSE and Activities of Daily Living [ADL])” (p.141) |
|  | Wang et al., 2021 | “psychosocial, such as improving QOL, restoring normal performance, maintaining mental health, improving adaptability (…) better adjustment (…) enhancing mental well-being (…) reducing care dependence, good social relations, positive self-image (..) reducing burden or stress (…) enhancing intent or meaning of life, and obtaining self-esteem, positive emotions, self-efficacy, boldness, active coping, optimism, social support, adaptation, and cognitive flexibility (including positive reassessment and acceptance)” (p.2) |
|  | Shoesmith et al., 2023 | “depression” (p.678), “quality of life” (p.678), “agitation” (p.678), “social connections” (p.679) |
|  | Clark et al., 2004 | “satisfaction with quality of (…) services, (…), depression, (…) perceived strain because of (…) memory problems, (…) difficulty in patients’ relationships with their caregivers (…) embarrassment and isolation as a result of memory problems (…) difficulty coping with memory problems” (p.43-44) |
|  | Möhler et al., 2023 | “agitation or challenging behaviour (…), quality of life (…) adverse effects (…) affect (…) mood (…) level of engagement (…) other dementia-related symptoms such as sleep disturbances, hallucinations or delusions (..) use of psychotropic medication” (p.7) |
|  | Zimmerman et al., 2013 | “Psychosocial outcomes for people with dementia included positive and negative affect (e.g., pleasure and anxiety); behavioral symptoms (e.g., agitation); engagement; quality of life; quality of dying; spiritual well-being; control, autonomy, and choice; satisfaction; and use of psychoactive medications and restraints.” (p.1401) |
|  | Ha et al., 2021 | “psychosocial outcomes—i.e., depressive symptoms, talkativeness, mutuality (for both caregivers and care recipients)” (p.832) |
|  | Feast et al., 2020b | “Psychosocial (comfort,well-being, a quality of life, social interaction etc)” (p.465), “pleasure and alertness (…) relaxation, distraction, engagement, and agitation” (p.483) |
|  | Brandt et al., 2005 | “observable psychosocial (patient anxiety (…) domains” (p.340) |
|  | Kumar and Salinas, 2021 | “Psychosocial outcomes (…) depression, anxiety, and stress” (p.7 of 13) |
| Psychosocial status | Wang et al., 2022 | “psychosocial status (quality of life)” (p.884) |
| Psychosocial health | Lakhani et al., 2019 | “psychosocial health (…) consist of psychological health (…), emotional health (…) and social health (…) social and emotional wellbeing, social reintegration and psychological adjustment” (p.188) |
| Psychosocial functioning | Shim et al., 2021 | “psychosocial functioning (e.g. anxiety, depression, worry, quality of life)” (p.545) “other psychosocial outcomes (avoidance, depression, confusion, fatigue, vitality, mental and physical functioning, and relationship quality)” (p.545) |
|  | Sidani et al., 2012 | “Residents’ physical and psychosocial functioning was measured with two complementary instruments: the Modified Interaction Behavior Measure (MIBM) and the London Psychogeriatric Rating Scale (LPRS). The MIBM was administered by the research assistant (RA) and focused on assessing  residents’ psychosocial functioning during morning care (…)The LPRS assesses residents’ mental  confusion, physical disability, socially irritating behaviour and disengagement” (p.40) |
|  | Kok et al., 2013 | “psychosocial function: MOSES scale” (Table 1, p.364) |
|  | Toseland et al., 1997 | “self-care, disorientation, depression, irritability, and withdrawal” (p.40) |
| Psychosocial wellbeing | Watson et al., 1998 | "depression/anxiety, irritability, withdrawal, disorientation" (p.300) |
|  | Rababa et al., 2023 | “Psychosocial wellbeing”, “the psychosocial and behavioral outcomes of PWD” (p.1), “behavioral health measures”, “aggression”, “agitation”, “anxiety”, “depression”, “isolation”, “lonely”, “fatigue”, “hopelessness”, “indifference”, “quality of life”, “positive and negative affectivity” (p.5-9) |
|  | Dawson et al., 2013 | “psychosocial well-being, such as anxiety, depression, and QoL[quality of life]” (p.749) |
|  | Fauth et al., 2020 | "lack of positive affect,or displaying no affect during periods of wakefulness, may, in fact, represent marginal or low levels of psychosocial well-being." (p.760) |
| Psychosocial symptoms | Lassell et al., 2022 | “psychosocial symptoms of dementia (BPSD [behavioural and psychological symptoms of depression]) (e.g. agitation)” (p.2135) |
| Psychosocial benefits | Brancatisano et al., 2020 | “psychosocial benefits, namely enhanced mood, relationship quality, greater acceptance of the dementia diagnosis” (p.608) |
| Psychosocial support | Van der Steen et al., 2017 | “Domain 8. Psychosocial and spiritual support (2/4 recommendations): 8.3 Religious activities, such as rituals, songs, and services may help the patient because these may be recognized even in severe dementia. 8.4 For dying people, a comfortable environment is desirable.” (p.5 of 14) |
| Psychosocial problems | Ausserhofer et al., 2016 | “psychosocial problems of residents, such as loneliness, boredom, helplessness, and lack of meaning”, “psychosocial (eg, cognitive status, mood, behavior, social activities, quality of life) outcomes” (p.687) |
|  | Vespa et al., 2002 | “A reduction of symptoms defined by us as antisocial is considered an index of reduction of stress and of ‘psychosocial’ problems. (…) The chosen test for the assessment of ‘antisocial’ behavior is the following: Cohen-Mansfield Agitation Inventory” (p.3) |
| Psychosocial difficulties | Cieza et al., 2015a | “psychosocial difficulties (PSDs), such as sleep disturbances, emotional instability and difficulties in personal interactions” (p.1), “Psychosocial difficulties (PSDs), such as sleep disturbances, emotional instability and difficulties in personal interactions and in work” (p.2) |
|  | Cieza et al., 2015b | “psychosocial difficulties (PSDs), such as sleep and memory problems and difficulties in maintaining relationships” (p.2), “valid and reliable metric with which information directly collected from persons with brain disorders (…) PARADISE 24” (p.9), “energy and drive”, “motivation”, “appetite”, “sleep functions”, “attention functions”, “memory functions”, “psychomotor functions”, “agitation & aggression”, “depressive mood”, “worry and anxiety”, “stress”, “executive functions”, “pain”, “sexual functions”, “communication”, “walking”, “self-care”, “independency in everyday activities”, “looking after one’s health”, “informal relationships with friends”, “family relationships and intimate relationships”, “education/work and employment”, “economic self-sufficiency”, “community, social and civic life” (Table 2) |
|  | Sabariego et al., 2015 | “psychosocial difficulties (PSD) in daily life, e.g. problems with managing daily routine or emotional lability” (p.1), “psychosocial difficulties (PSD), ranging from problems with attention and memory, emotional lability and listlessness to problems with managing their daily routines, problems interacting with significant others and difficulties at work” (p.2) |

Notes: p, page.

**Table S3**. Outcome measures reported on, by psychosocial wellbeing domain.

| Psychosocial Wellbeing Domain | Measurement Instruments Used |
| --- | --- |
| General wellbeing | - Quality of Life-Alzheimer's Disease (QoL-AD) scale (Bourne et al., 2021, Wang et al., 2021, Shoesmith et al., 2023, Möhler et al., 2023, Shim et al., 2021, Dawson et al., 2013) - Dementia Quality of Life Measure (DEM-QOL) (Bourne et al., 2021, Wang et al., 2021) - Dementia Quality of Life Proxy Measure (DemQOL-Proxy) (Wang et al., 2021, Bourne et al., 2021) - European Quality of Life 5 Dimensions (EQ-5D) (Miao et al., 2021) - Short-Form-8 (SF-8) (Bourne et al., 2021) - Blau Quality of Life Scale (Wang et al., 2022) - World Health Organization Quality-of-Life Scale (WHO-QoL) (Bourne et al., 2021, Shim et al., 2021) - Alzheimer’s Disease Rated Quality of Life-40 item (ADRQL-40) (Wang et al., 2021) - Quality of life in late-stage dementia (QUALID) (Shoesmith et al., 2023) - Dementia Quality of Life instrument (DQOL) (Wang et al., 2021) - World Health Organisation Well-being Index (WHO-5) (Wang et al., 2021) - Standard Quality Assessment Criteria (Bourne et al., 2021) - Quality of Life (MCS-SF36) (Shim et al., 2021) - Quality of Life (AQol-8D) (Shim et al., 2021) - Instant Assessment of Wellbeing Tool (EVIBE) (Feast et al., 2020a) - Psychological Wellbeing Scale (PWS) (Shim et al., 2021) - Palliative Care Outcome Scale (POS) (Hermans et al., 2017, Brandt et al., 2005) - Visual Analogue Scale for Wellbeing (Bourne et al., 2021) - Study-specific multi-dimensional measure (Clark et al., 2004) - Spirituality Index of Well-Being (Yen and Lin, 2018) - Life Satisfaction Index-A (LSI-A) (Yen and Lin, 2018) |
| Emotional wellbeing | - Center for Epidemiological Studies Depression scale (CES-D) (Clark et al., 2004, Dawson et al., 2013, Shim et al., 2021) - Cornell Scale for Depression in Dementia (CSDD) (Resnick and Galik, 2015b, Shoesmith et al., 2023, Wang et al., 2021, Möhler et al., 2023, Wang et al., 2022) - Geriatric Depression Scale (GDS) (Ha et al., 2021, Wang et al., 2021, Möhler et al., 2023, Wang et al., 2022, Yen and Lin, 2018, Lee et al., 2019) - Multi Dimensional Observation Scale for Elderly Subjects (MOSES) (Toseland et al., 1997, Watson et al., 1998, Kok et al., 2013) - Palliative Care Outcome Scale (POS) (Brandt et al., 2005, Hermans et al., 2017) - The Philadelphia Geriatric Center Positive and Negative Affect Rating Scale (Fauth et al., 2020) - International Positive and Negative Affect Schedule Short-Form (I-PANAS-SF) (Feast et al., 2020a) - Nurses’ Observation Scale for Geriatric Patients - subscale mood (NOSGER) (Wang et al., 2021) - Dementia-specific quality of life (QUALIDEM)-subscales on affect (Wang et al., 2021) - PARADISE Data Collection Protocol (Cieza et al., 2015a, Sabariego et al., 2015) - Geriatric Depression Scale (GDS) (Ha et al., 2021, Shim et al., 2021, Shoesmith et al., 2023) - Dementia Mood Assessment Scale (DMAS) (Shoesmith et al., 2023) - Depression Anxiety Stress Scales (DSS) (Bourne et al., 2021) - Taiwanese Depression Questionnaire (Lee et al., 2019) - Zung Self-Rating Anxiety Scale (Z-SAS) (Dawson et al., 2013) - Rating Anxiety In Dementia (RAID) (Wang et al., 2021, Möhler et al., 2023, Wang et al., 2022) - Geriatric Anxiety Inventory (GAI) (Shim et al., 2021) - Observed emotion rating scale (Shoesmith et al., 2023) - Lawton Observed Emotion Rating Scale (OERS) (Feast et al., 2020b, Möhler et al., 2023) - Arts Observational Scale (ArtsObs) (Feast et al., 2020a) - Philadelphia Geriatric Center Affect Rating Scale (ARS) (Möhler et al., 2023) - Apparent Affect Rating Scale (AARS) (Kok et al., 2013) - Mood Picture Test (Möhler et al., 2023) - Palliative Care Outcome Scale (POS) (Hermans et al., 2017, Brandt et al., 2005) - Mood and Feelings Questionnaire (MFQ) (Shim et al., 2021) - Perceived Stress Scale (PSS) (Wang et al., 2021) - Observable Displays of Affect Scale (Lee et al., 2019) - Apparent Affect Rating Scale (Lee et al., 2019) - P.I.E.C.E.S. framework (Timmons and Fox, 2023) - Herth Hope Index (Yen and Lin, 2018) - Worry Questionnaire for Continuing Care Residents (Lee et al., 2019) - Study-Specific Embarrassment Measure (Clark et al., 2004) - Needs Assessment (Bowen et al., 2014) - Qualitative interviews (Schmidt et al., 2018, Damianakis et al., 2010) |
| Cognitive functioning | - Blessed Test (Clark et al., 2004) - Alzheimer's Disease Assessment Scale - cognitive subscale (ADAS-Cog) (Bourne et al., 2021) - The Trail Making Test (TMT) (Wang et al., 2022) - Montreal Cognitive Assessment Scale (MoCa) (Wang et al., 2022) - The World Health Organisation-University of California Los Angeles-Auditory Verbal Learning (WHO-UCLA-AVLT) (Wang et al., 2022) - Mini-Mental State Examination (MMSE) (Wang et al., 2022, Feast et al., 2020a, Yen and Lin, 2018, Shoesmith et al., 2023, Bourne et al., 2021) - Clinical Dementia Rating (CDR) Scale (Yen and Lin, 2018, Timmons and Fox, 2023) - Global Deterioration Scale (Bourne et al., 2021) - PARADISE Data Collection Protocol (Sabariego et al., 2015, Cieza et al., 2015b) - Clinical Dementia Rating Scale (CDR) (Bourne et al., 2021) - Addenbrookes Cognitive Examination – Revised (ACE-R) (Bourne et al., 2021) - Severe Impairment Strategy (SIB) (Shoesmith et al., 2023) |
| Physical health | - Needs Assessment (Bowen et al., 2014) - Berg Balance Scale (Lee et al., 2019) - Physical Activity Survey in Long-Term Care (PASLTC) (Lee et al., 2019) - Tinnetti Scale (Lee et al., 2019, Feast et al., 2020a) - Short Form-36 (SF-36) (Shoesmith et al., 2023) - Health-related quality of life (Shim et al., 2021) - Time Up and Go (TUG) Test (Shoesmith et al., 2023, Wang et al., 2022) - Short Physical Performance Battery (SPPB) (Wang et al., 2022) - Step Test (Wang et al., 2022) - Timed-Chair-Stand Test (Wang et al., 2022) - Functional Reach Test (Wang et al., 2022) - Mini Physical Performance Test (mPPT) (Wang et al., 2022) |
| Behavioural Symptoms | - Cohen-Mansfield Agitation Inventory (CMAI) (Watson et al., 1998, Toseland et al., 1997, Resnick and Galik, 2015a, Möhler et al., 2023, Shoesmith et al., 2023, Kok et al., 2013, Wang et al., 2022) - Brief Agitation Rating Scale (Shoesmith et al., 2023, Wang et al., 2021) - Pittsburgh Agitation Scale (Feast et al., 2020a, Wang et al., 2021, Wang et al., 2022) - PARADISE Data Collection Protocol (Cieza et al., 2015a, Cieza et al., 2015b ) - Multi Dimensional Observation Scale for Elderly Subjects (MOSES) (Toseland et al., 1997, Watson et al., 1998, Kok et al., 2013) - Apathy Evaluation Scale (Resnick and Galik, 2015b) - Neuropsychiatric Inventory (NPI) (Kok et al., 2013, Shoesmith et al., 2023, Wang et al., 2021, Lee et al., 2019, Bourne et al., 2021, Wang et al., 2022) - Long-Term Care Resident Assessment Instrument (LRAI) (Kok et al., 2013) - Revised Memory and Behaviour Problems Checklist (RMBPC) (Kok et al., 2013) - Zimmerman’s short version of Apathy Evaluation Scale (AES) (Shoesmith et al., 2023) - Agitated Behaviors Mapping Instrument (AGMI) (Shoesmith et al., 2023, Möhler et al., 2023) - Minimum data set 2.0 section E4 (Kok et al., 2013) - Nursing Home Behavioral Problem Scale (NHBPS) (Kok et al., 2013, Shoesmith et al., 2023) - Alzheimer’s Disease Assessment Scale (ADAS) (Kok et al., 2013) - Adaptive Behavior Rating Scale (ABRS) (Kok et al., 2013) - Behavior Rating Form (BRF) (Kok et al., 2013) - Agitated Behaviors in Dementia scale (ABID) (Wang et al., 2021) - Pittsburgh Sleep Quality Index (PSQI) (Wang et al., 2022, Shim et al., 2021) - Institutional Comprehensive Assessment and Referral Evaluation (INCARE) (Kok et al., 2013) - Delirium Severity Scale (Feast et al., 2020a) - Passivity in Dementia Scale (PDS) (Möhler et al., 2023) - Disruptive Behavior Scale (Lee et al., 2019) - Exposure to Aggression during Caregiving subscale (Kok et al., 2013) |
| Daily Life Functioning | - Barthel Index (Resnick and Galik, 2015b, Lee et al., 2019, Shoesmith et al., 2023) - Bristols Activities of Daily Living Scale (Bourne et al., 2021) - Clinical Dementia Rating Scale (CDR) (Bourne et al., 2021) - Katz Index of Activities of Daily Living (KI-ADL) (Feast et al., 2020a) - Refined Activities of Daily Living Assessment Scale (RADL) (Feast et al., 2020a) - Beck Dressing Performance Scale (BDP) (Feast et al., 2020a) - Global Deterioration Scale (Bourne et al., 2021) - PARADISE Data Collection Protocol (Sabariego et al., 2015, Cieza et al., 2015b) |
| Self | - Palliative Care Outcome Scale (POS) (Hermans et al., 2017, Brandt et al., 2005) - Rosenberg Self-Esteem Scale (RSES) (Lee et al., 2019, Bourne et al., 2021) |
| Caregiving | - Needs Assessment (Bowen et al., 2014) - Caregiving burden (Ha et al., 2021) - Zarit Burden Interview (ZBI) (Bourne et al., 2021) - Caregivers' quality of life (Carer-QoL) (Shoesmith et al., 2023) - Family Caregiving Inventory (Shoesmith et al., 2023) - Preparedness for Caregiving Scale (Feast et al., 2020a) - Hospital Anxiety and Depression Scale (HADS) (Feast et al., 2020a) - Confidence in Dementia Scale (CODE) (Feast et al., 2020a) - Knowledge in Dementia (KIDE) (Feast et al., 2020a) - Approaches to Dementia Questionnaire (ADQ) (Feast et al., 2020a) - Alzheimer's Disease Knowledge Scale (ADKS) (Feast et al., 2020a) - Dementia Symptom Knowledge Assessment (Shim et al., 2021) - Dementia Knowledge Scale (DK-20) (Feast et al., 2020a) - Sense of Competency in Dementia Care (SCIDS) (Feast et al., 2020a) |
| Coping with Dementia | - Study-Specific Measure of Embarrassment, Isolation and Coping with Memory Problems (Clark et al., 2004) |
| Medical Treatment | - Needs Assessment (Bowen et al., 2014) - Study-specific Service Satisfaction Scale (Clark et al., 2004) - Satisfaction with Care at End-of-Life in Dementia (Shim et al., 2021) - Clinical Outcome Variables (S-COVS) (Feast et al., 2020a) |
| Social Health | - Social Activities (Kok et al., 2013) - ‘Antisocial’ Behavior with the Cohen-Mansfield Agitation Inventory (Vespa et al., 2002) - Qualitative Interview (Schmidt et al., 2018) - Four-Item Index of Relationship Strain (Clark et al., 2004) - Caregiver Burden Questionnaire (Ha et al., 2021) - Dementia-specific quality of life (QUALIDEM) - subscale care relationship (Wang et al., 2021) - London Psychogeriatric Rating Scale (LPRS) (Sidani et al., 2012) - Palliative Care Outcome Scale (POS) (Timmons and Fox, 2023, Hermans et al., 2017) - Nurses’ Observation Scale for Geriatric Patients (NOSGER) (Wang et al., 2021) - Modified Interaction Behavior Measure (MIBM) (Sidani et al., 2012) - PARADISE Data Collection Protocol (Sabariego et al., 2015, Cieza et al., 2015a, Cieza et al., 2015b) - Medical Outcomes Study social support survey (MOS) (Bourne et al., 2021) - Neuropsychiatric Inventory (NPI) (Kok et al., 2013) - Institutional Comprehensive Assessment and Referral Evaluation (INCARE) (Kok et al., 2013) - Index of Social Engagement (Möhler et al., 2023) - Revised Index of Social Engagement from the Resident Assessment Instrument (RISE) (Kok et al., 2013) - Multi Dimensional Observation Scale for Elderly Subjects (MOSES) (Kok et al., 2013) - Pleasant Events Scale (Kok et al., 2013) - Multifocus Assessment Scale-Revised (MAS-R) (Kok et al., 2013) - Mutuality Scale (Ha et al., 2021) - Talkativeness Scale (Ha et al., 2021) - Study-Specific Isolation Measure (Clark et al., 2004) - Qualitative Interview (Schmidt et al., 2018, Damianakis et al., 2010, Van der Steen et al., 2017) - Family Assessment Measure (FAM) (Bourne et al., 2021) - Social Behaviour Observation Checklist (Shoesmith et al., 2023) |
| Activities | - Integrated Palliative care Outcome Scale for Dementia (IPOS-Dem) (Timmons and Fox, 2023) - Menorah Park Engagement Scale (MPES) (Möhler et al., 2023, Zimmerman et al., 2013, Shoesmith et al., 2023, Feast et al., 2020a) - Myers Research Institute Engagement Scale (MRI-ES) (Möhler et al., 2023) - Observational scale measuring mood, distraction, and relaxation (ArtsObs) (Zimmerman et al., 2013) |

**Table S4.** Description of included records with empirical evidence, by terminology used for psychosocial wellbeing.

| **Publication** | **Record type** | **Population** | **Setting** | **Intervention** | **Comparison** | **Psychosocial outcome** | **Significant findings** | **Quality rating** |
| --- | --- | --- | --- | --- | --- | --- | --- | --- |
| **Psychosocial needs** |  |  |  |  |  |  |  |  |
| Bowen et al., 2014 | Cross-sectional (Level 6) | n=204 PwD, Age 25-97 (M: 69) | Outpatient clinics | None | None | Psychosocial Needs Assessment (incl. care network, living  situation, safety, physical state, level of independence, activities of daily living, caregiver education, available  resources and services) | 52.5% assistance driving, 49.0% assistance administering medication, 42.2% assistance managing finances, 37.8% assistance shopping, 36.3% want exercise, 35.8% assistance meal preparation, 31.8 % <6 h sleep, 27.5% nutritional guidance, 23.5% negative mood, 21.6% assistance bathing, 19.6% assistance dressing. 10.8% safety concerns, 8.3% high pain | Good |
| Lee et al., 2019 | Review (Level 5) | n=9,830 older adults (mostly PwD), Age M: 85 | Nursing Homes | Function-Focused Care | Mixed | Psychosocial function (e.g. self-esteem, communication, autonomy), cognitive function, physical function^1^ | 86% of studies significant improvements in mood, affect, behavioural problems, movement, balance, and activities of daily living | Good |
| Schmidt et al., 2018 | Qualitative (Level 6) | n=30 PwD, Age 75–93 (M: 84) + n=41 health professionals and n=12 relatives | Palliative care homes | None | None | Needs (group discussions, interviews) | Total of 25 needs in 10 categories: food intake, physical well-being. physical activity and recovery, adaptation of stimuli, communication, personal attention, participation, familiarity and safety, self-determination, religion | High |
| Hermans et al., 2017 | Cross-sectional (Level 6) | n=60 PwD, n=49 older adults without dementia, Age 65-90+ (M: 87) | Palliative care homes | None | None | Palliative Care Outcome Scale (POS; incl. patient anxiety, support, finding life worth living, self-worth) | PwD (vs. non-PwD) fewer physical symptoms apart from pain (b=–0.73, p=0.001), higher needs in support (b=0.75, p=0.015), life worthwhile (b=0.58, p=0.020) and self-worth (b=0.58, p=0.012) | High |
| **Psychosocial outcomes** |  |  |  |  |  |  |  |  |
| Resnick and Galik, 2015a | Cohort (Level 4) | n=199 PwD, Age 58-105 (M: 85) | Nursing homes, assisted living | Function-focused care in residential care vs. nursing home | None | Cornell Scale for Depression in Dementia, Cohen-Mansfield Agitation Inventory (CMAI) short form, Apathy Evaluation Scale,  Physical activity (counts, minutes, calories),^1^ Barthel index (daily functioning)^1^ | Nursing home residents (compared to residential) had increased calories (F=4.01; p=0.002) and better daily functioning (Barthel-index F=6.38, p=0.001) | High |
| Bourne et al., 2021 | Review (Level 5) | n=169 PwD, Age 57-91 (M: 78) | Mixed | Dyadic Art Intervention | Mixed | Psychosocial outcomes (i.e. emotions, wellbeing, QoL, behaviour, forming and maintaining connections with others) | Improved well-being, quality of life, mood, enhanced identity, decreased social isolation | Good |
| Damianakis et al., 2010 | Qualitative (Level 6) | n=6 PwD, n=6 MCI, Age 60-95 (M: 80) | Participants’ homes | Multimedia Biography Screenings | None | Mood, memory, caregiver perceptions (interviews, video recordings) | Reports on stimulated reminiscing, positive mood response, increased understanding, and improved social interactions | Good |
| Wang et al., 2021 | Review (Level 1) | n=2,442 with neurocognitive disorders (mostly dementia) | Mixed | Resilience Intervention | Mixed | Psychosocial outcomes (e.g. QoL, social relations, positive  self-image, self-efficacy, hardiness, anxiety, depression) | Improved QoL (SMD=0.14, p=0.02) | High |
| Shoesmith et al., 2023 | Review (Level 5) | n=1,841 PwD | Mixed | Animal-assisted, robotic animal interventions | Mixed | Agitation, depression, quality of life | Reduced agitation (SMD=0.74, p=0.003) | Good |
| Clark et al., 2004 | RCT (Level 2) | n=121 PwD, Age 55+ (M: 76-78) | Participants’ homes | Telephone care consultation | Care-as-usual | Center for  Epidemiological Studies Depression (CES-D) scale items, Four-Item Index of Relationship Strain, embarrassment, isolation, coping difficulties, Blessed Test (cognition) | Decreased depression for those with more memory difficulties (b=-0.33; p<0.07), decreased relationship strain (b=-0.01, p<0.05), decreased feelings of embarrassment and isolation (b=-0.17; p<0.07), decreased difficulty coping (b=-0.22, p<0.05). | Good |
| Möhler et al., 2023 | Review (Level 1) | n=1,071 PwD, Age M: 78-88 | Nursing homes | Personally tailored activities | Mixed | Agitation, QoL, affect, mood, engagement, BPSDs, use of psychotropic medication | Reduced agitation (SMD=0.26, p=0.01) | High |
| Zimmerman et al., 2013 | Review (Level 5) | n=4,746+ older adults (at least 80 % PwD) | Long-term care | Characteristics of long-term care settings | Mixed | Positive and negative affect, BPSDs, engagement, QoL, quality of dying, spiritual well-being, control, autonomy, choice, satisfaction, use of psychoactive medications and restraints | Pleasant sensory stimulation: reduced agitation; individualized care: better outcomes (function, quality of life, behavioural symptoms,  pain and discomfort) | Good |
| Ha et al., 2021 | RCT (Level 3) | n=37 PwD, Age M: 76 | Participants’ homes | Couples Lifestory Approach | None | Geriatric Depression Scale (GDS), talkativeness, mutuality, caregiver burden | Talkativeness declined among female PwD but increased among male PwD | Good |
| Feast et al., 2020b | Review (Level 5) | n=4,036 PwD, Age M: 79, M | Hospitals | Hospital care interventions | Mixed | Comfort, wellbeing, QoL, social interaction, other psychosocial outcomes, BPSDs^1^, physical function^1^ | Very low quality evidence of improved BPSDs | Good |
| Brandt et al., 2005 | Cross-sectional  (Level 6) | n=302 PwD, n=146 other palliative patients, Age 45-100 (M: 84) | Palliative care homes | None | None | Palliative Care Outcome Scale (POS; incl. patient anxiety, support, finding life worth living, self-worth) | PwD (vs other palliative patients) less able to share feelings (i.e., more support needs; M: 1.5 vs. M: 2.0, p=0.030) | Good |
| **Psychosocial status** |  |  |  |  |  |  |  |  |
| Wang et al., 2022 | Review (Level 1) | n=583 PwD, Age M: 82 | Mixed | Mind-body therapies | Mixed | Psychosocial status (QoL), Neuropsychiatric Inventory (NPI; i.e. BPSDs),^1^ Mini Mental State Examination (MMSE; i.e. cognitive function),^1^ depression^1^, agitation,^1^ anxiety^1^, Timed Up and Go test (TUG) and Barthel Index ( i.e. physical function)^1^ | Tai Chi improved Mini-Mental State Examination (SMD=0.40, p=0.01), Yoga and aromatherapy may improve depression, all may improve quality of life | Good |
| **Psychosocial health** |  |  |  |  |  |  |  |  |
| Lakhani et al., 2019 | Review (Level 5) | People with neurocognitive disorders (majority PwD) | Mixed | Natural environment interventions | Mixed | Psychological, social, and emotional health | Improved psychological health (50% quantitative studies, one qualitative study); improved social health (40% quantitative studies, three qualitative studies; improved emotional health (58.3% quantitative studies, one qualitative study) | Good |
| **Psychosocial functioning** |  |  |  |  |  |  |  |  |
| Shim et al., 2021 | Review (Level 1) | n=530 people in pre-stages of and with dementia, Age M: 72 | Mixed | Mindfulness-based interventions | Mixed | Psychosocial functioning (e.g. anxiety, depression, worry, QoL) | overall results inconsistent, indicate post-treatment effects in medium to large range for psychosocial outcomes,  small to medium range for cognitive functioning | Good |
| Toseland et al., 1997 | RCT (Level 3) | n= 88 PwD, Age M: 88 | Nursing homes | Validation group therapy | Social Contact, Care-as-usual | Multidimenisonal Observation Scale for Elderly Subjects (MOSES; incl. self-care, disorientation, depression, irritability, and withdrawal), Cohen-Mansfield Agitation Inventory (CMAI),^1^ Geriatric Indices of Positive Behaviour,^1^ Minimum Data Set Resident Assessment Protocol (incl. medication received, use of restraints)^1^ | MOSES: improved score F(30,706)=1.41, p<0.05), CMAI: reduced aggression baseline to 1-year (x2=14.90, p=0.001), reduced non-aggression baseline to 1-year (x2=6.76, p=0.034), reduced verbal aggression baseline to 1-year (x2=12.46, p=0.002) | Good |
| **Psychosocial wellbeing** |  |  |  |  |  |  |  |  |
| Watson et al., 1998 | RCT (Level 2) | n=25 PwD, Age 72-95 (M: 83) | Nursing homes | Rocking chair intervention | Crossover | Multidimensional Observation Scale for Elderly Subjects (MOSES; incl. self-care, disorientation, depression, irritability, and withdrawal), Cohen-Mansfield Agitation Inventory (CMAI),^1^ nursing home records of incidents/accidents/use of pain medication^1^, balance^1^ | MOSES: reduced anxiety/ depression (t=-2.06, p=0.026); reduced pain medication (t=-4.04, p<0.001) | Good |
| Dawson et al., 2013 | Cross-sectional (Level 6) | n=131 PwD, Age 50-95 (M: 77) | Participants’ homes | None | None | Zung Self-Rating Anxiety Scale (Z-SAS), Center for Epidemiologic Studies Depression Scale (CES-D), Quality of Life in Alzheimer's Disease (QoL-AD) scale | embarrassment about memory problems (β=0.22, p<0.05) with anxiety; role captivity relationship strain (β=0.23, p<0.01)/ physical health strain (β=0.36, p<0.001) with depression; difficulty with IADLs (β=-0.28, p<0.01)/ inner strength/growth self-efficacy perception (β=0.30, p<0.001) with quality of life | High |
| Fauth et al., 2020 | Cross-sectional (Level 6) | n=22 PwD, Age 49-93 (M: 76) | Nursing home | Staff interactions | None | The Philadelphia Geriatric Center Positive and Negative Affect Rating Scale | Enhanced positive affect (81% of the time, z=28.84, p<0.001) | Good |
| **Psychosocial symptoms** |  |  |  |  |  |  |  |  |
| Lassell et al., 2022 | Review (Level 5) | PwD in any hospice setting, Age 80-89 | Hospice | Any hospice interventions | Mixed | BPSDs, quality of life, pain assessment | Improved pain (p≤0.05), agitation (p<0.001), quality of life (p=0.001); one study: improved neuropsychiatric symptom severity (p≤0.05) | Good |

Notes: BPSDs: behavioural and psychological symptoms of dementia; M, mean; PwD, people with dementia; QoL: quality of life; RCT, randomized controlled trial; SMD, standardized mean difference.

^1^outcome not included in the authors’ definition of ‘psychosocial wellbeing’ nonetheless listed here as it matches the definition identified in review

**References**

**Ausserhofer, D.*, et al.*** 2016. “There's no place like home”: a scoping review on the impact of homelike residential care models on resident-, family-, and staff-related outcomes. *Journal of the American Medical Directors Association,* 17**,** 685-693.

**Bourne, P., Camic, P. M. & Crutch, S. J.** 2021. Psychosocial outcomes of dyadic arts interventions for people with a dementia and their informal caregivers: A systematic review. *Health & Social Care in the Community,* 29**,** 1632-1649.

**Bowen, K. J., Gonzalez, E. W., Edwards, C. Y. & Lippa, C. F.** 2014. Needs assessments of memory disorder patients. *Am J Alzheimers Dis Other Demen,* 29**,** 667-72.

**Brancatisano, O., Baird, A. & Thompson, W. F.** 2020. Why is music therapeutic for neurological disorders? The Therapeutic Music Capacities Model. *Neuroscience & Biobehavioral Reviews,* 112**,** 600-615.

**Brandt, H. E.*, et al.*** 2005. The last days of life of nursing home patients with and without dementia assessed with the palliative care outcome scale. *Palliat Med,* 19**,** 334-42.

**Cieza, A.*, et al.*** 2015a. Understanding the impact of brain disorders: towards a ‘horizontal epidemiology’of psychosocial difficulties and their determinants. *PLoS One,* 10**,** e0136271.

**Cieza, A.*, et al.*** 2015b. PARADISE 24: a measure to assess the impact of brain disorders on people’s lives. *PloS one,* 10**,** e0132410.

**Clark, P. A., Bass, D. M., Looman, W. J., Mccarthy, C. A. & Eckert, S.** 2004. Outcomes for patients with dementia from the Cleveland Alzheimer's Managed Care Demonstration. *Aging & Mental Health,* 8**,** 40-51.

**Damianakis, T., Crete-Nishihata, M., Smith, K. L., Baecker, R. M. & Marziali, E.** 2010. The psychosocial impacts of multimedia biographies on persons with cognitive impairments. *Gerontologist,* 50**,** 23-35.

**Dawson, N. T., Powers, S. M., Krestar, M., Yarry, S. J. & Judge, K. S.** 2013. Predictors of self-reported psychosocial outcomes in individuals with dementia. *Gerontologist,* 53**,** 748-59.

**Fauth, E. B., Meyer, K. V. & Rose, C.** 2020. Co-occurrence of positive staff interactions and positive affect in memory-care residents: An observational study. *Int J Geriatr Psychiatry,* 35**,** 759-768.

**Feast, A. R., White, N., Candy, B., Kupeli, N. & Sampson, E. L.** 2020a. The effectiveness of interventions to improve the care and management of people with dementia in general hospitals: A systematic review. *Int J Geriatr Psychiatry,* 35**,** 463-488.

**Feast, A. R., White, N., Candy, B., Kupeli, N. & Sampson, E. L.** 2020b. The effectiveness of interventions to improve the care and management of people with dementia in general hospitals: A systematic review. *International Journal of Geriatric Psychiatry,* 35**,** 463-488.

**Ha, J.-H.*, et al.*** 2021. The effectiveness of a couple-based intervention for people living with mild Alzheimer’s disease and their spousal caregivers in Korea. *Dementia,* 20**,** 831-847.

**Hermans, K., Cohen, J., Spruytte, N., Van Audenhove, C. & Declercq, A.** 2017. Palliative care needs and symptoms of nursing home residents with and without dementia: A cross-sectional study. *Geriatr Gerontol Int,* 17**,** 1501-1507.

**Kok, J. S., Berg, I. J. & Scherder, E. J.** 2013. Special care units and traditional care in dementia: relationship with behavior, cognition, functional status and quality of life-a review. *Dementia and geriatric cognitive disorders extra,* 3**,** 360-375.

**Kumar, A. & Salinas, J.** 2021. The long-term public health impact of social distancing on brain health: topical review. *International journal of environmental research and public health,* 18**,** 7307.

**Lakhani, A., Norwood, M., Watling, D. P., Zeeman, H. & Kendall, E.** 2019. Using the natural environment to address the psychosocial impact of neurological disability: A systematic review. *Health Place,* 55**,** 188-201.

**Lassell, R. K. F.*, et al.*** 2022. Hospice interventions for persons living with dementia, family members and clinicians: A systematic review. *J Am Geriatr Soc,* 70**,** 2134-2145.

**Lee, S. J., Kim, M. S., Jung, Y. J. & Chang, S. O.** 2019. The Effectiveness of Function-Focused Care Interventions in Nursing Homes: A Systematic Review. *J Nurs Res,* 27**,** 1-13.

**Miao, L.*, et al.*** 2021. The influence of music tempo on mental load and hazard perception of novice drivers. *Accident Analysis & Prevention,* 157**,** 106168.

**Möhler, R., Calo, S., Renom, A., Renom, H. & Meyer, G.** 2023. Personally tailored activities for improving psychosocial outcomes for people with dementia in long‐term care. *Cochrane Database of Systematic Reviews*.

**Rababa, M., Aldrawsheh, A., Hayajneh, A. A. & Da’seh, A.** 2023. Environmental and Caregivers-Related Factors Influencing the Psychosocial Well-Being of Older Adults with Dementia: A Systematic Review. *Ageing International,* 48**,** 999-1010.

**Resnick, B. & Galik, E.** 2015a. Impact of care settings on residents' functional and psychosocial status, physical activity and adverse events. *International Journal of Older People Nursing,* 10**,** 273-283.

**Resnick, B. & Galik, E.** 2015b. Impact of care settings on residents' functional and psychosocial status, physical activity and adverse events. *Int J Older People Nurs,* 10**,** 273-83.

**Sabariego, C.*, et al.*** 2015. Determinants of psychosocial difficulties experienced by persons with brain disorders: towards a ‘horizontal epidemiology’approach. *PLoS One,* 10**,** e0141322.

**Schmidt, H., Eisenmann, Y., Golla, H., Voltz, R. & Perrar, K. M.** 2018. Needs of people with advanced dementia in their final phase of life: A multi-perspective qualitative study in nursing homes. *Palliat Med,* 32**,** 657-667.

**Shim, M., Tilley, J. L., Im, S., Price, K. & Gonzalez, A.** 2021. A Systematic Review of Mindfulness-Based Interventions for Patients with Mild Cognitive Impairment or Dementia and Caregivers. *Journal of Geriatric Psychiatry and Neurology,* 34**,** 528-554.

**Shoesmith, E., Surr, C. & Ratschen, E.** 2023. Animal-assisted and robotic animal-assisted interventions within dementia care: A systematic review. *Dementia,* 22**,** 664-693.

**Sidani, S., Streiner, D. & Leclerc, C.** 2012. Evaluating the effectiveness of the abilities‐focused approach to morning care of people with dementia. *International Journal of Older People Nursing,* 7**,** 37-45.

**Timmons, S. & Fox, S.** 2023. Palliative care for people with dementia. *Handbook of Clinical Neurology,* 191**,** 81-105.

**Toseland, R. W.*, et al.*** 1997. The Impact of Validation Group Therapy on Nursing Home Residents With Dementia. *Journal of Applied Gerontology,* 16**,** 31-50.

**Van Der Steen, J. T.*, et al.*** 2017. Palliative care for people with dementia in the terminal phase: a mixed-methods qualitative study to inform service development. *BMC palliative care,* 16**,** 1-14.

**Vespa, A., Gori, G. & Spazzafumo, L.** 2002. Evaluation of non-pharmacological intervention on antisocial behavior in patients suffering from Alzheimer's disease in a day care center. *Archives of gerontology and geriatrics,* 34**,** 1-8.

**Wang, Y., Chi, I., Zhan, Y., Chen, W. & Li, T.** 2021. Effectiveness of resilience interventions on psychosocial outcomes for persons with neurocognitive disorders: a systematic review and meta-analysis. *Frontiers in Psychiatry,* 12**,** 709860.

**Wang, Y., Tang, C., Fan, X., Shirai, K. & Dong, J.-Y.** 2022. Mind–body therapies for older adults with dementia: a systematic review and meta-analysis. *European Geriatric Medicine,* 13**,** 881-891.

**Watson, N. M., Wells, T. J. & Cox, C.** 1998. Rocking chair therapy for dementia patients: Its effect on psychosocial well-being and balance. *American Journal of Alzheimer's Disease,* 13**,** 296-308.

**Yen, H.-Y. & Lin, L.-J.** 2018. A systematic review of reminiscence therapy for older adults in Taiwan. *Journal of Nursing Research,* 26**,** 138-150.

**Zimmerman, S.*, et al.*** 2013. Systematic review: Effective characteristics of nursing homes and other residential long-term care settings for people with dementia. *J Am Geriatr Soc,* 61**,** 1399-409.
